# Alzheimer's Disease and Oral-Systemic Health: Bidirectional Care Integration Improving Outcomes

**DOI:** 10.3389/froh.2021.674329

**Published:** 2021-07-05

**Authors:** Anne O. Rice

**Affiliations:** Oral Systemic Seminars, Conroe, TX, United States

**Keywords:** Alzheimer's disease, medicare, sleep apnea, smoking, dentistry, pathogens, diabetes, cardiovascular disease

## Abstract

Dentistry is an effective healthcare field that can impact Alzheimer's disease through prevention and education. Every day dental providers use an arsenal of assessment protocols directly coinciding with modifiable Alzheimer's risk factors. An innovative way to help in the prevention of Alzheimer's disease is to utilize oral health professionals who reach the public in ways other health care providers may not. Bidirectional care integration is needed to stifle many systemic diseases and Alzheimer's disease is no different. Ultimately with collaborative care the patient reaps the benefits. Alzheimer's is associated with many etiologies and pathophysiological processes. These include cardiovascular health, smoking, sleep, inflammatory pathogens, and diabetes. In the United States, dental providers assess each of these factors daily and can be instrumental in educating patients on the influence of these factors for dementia prevention. Globally, by 2025, the number of people with Alzheimer's disease is expected to rise by at least 14%. Such increases will strain local and national health care systems, but for the US if Medicare were expanded to include dental services, many older adults could be spared needless suffering. The goal of this perspective article is to highlight existing practices being used in the field of dentistry that can easily be adapted to educate patients in preventive care and treat risk factors. It is the duty of healthcare professionals to explore all opportunities to stem the advance of this disease and by integrating oral and systemic health into transdisciplinary science, health care and policy may do just that.

## Introduction

Alzheimer's disease (AD) is a progressive mental deterioration that occurs in middle or old age due to the brain's degeneration [1]. Every 65 s, someone in the United States is diagnosed with Alzheimer's disease, the most common form of dementia, and it currently affects over 5.8 million people in the United States. The Alzheimer's Association projects by 2050 nearly 15 million people in the United States will receive an Alzheimer's diagnosis. The same report shows that a full half of primary care physicians feel unprepared to meet the demand of such a drastic increase [2]. In 2014, Rush University Medical Center analyzed death records nationally and found that most people who die from Alzheimer's have a recorded cause of death listed as respiratory or cardiac disease. When taking these numbers into account, AD would be elevated to the third leading cause of death in the United States [3].

Hundreds of billions of dollars in research and drug exploration have yielded a 99.6% drug failure rate [4]. While various drug developers are taking different approaches considering such near complete failure, many scientists and physicians are targeting prevention as the best strategy to reduce Alzheimer's cases [5–10]. In 2017, *The Lancet* Commission presented a report that stated more than one-third of global dementia cases may be preventable through addressing lifestyle factors that impact a person's risk [11]. The World Health Organization (WHO) agreed with this assessment and strongly recommended physical activity, quitting smoking, managing hypertension, and diabetes to reduce the risk of cognitive decline [12]. Disturbed sleeping patterns and periodontal disease have also been related to AD and accelerated cognitive decline rates [13–19].

The brain changes from Alzheimer's disease start at least a decade before symptoms show [20, 21], and as stated above, prevention is the best current defense against this disease. Prevention strategies should begin years before the typical onset presents, which is where dental providers can make the most significant contribution. Every day, dental professionals are performing vital tests, giving education on preventative measures, and prescribing medications in the line of treating and preventing dental health concerns that may also serve to reduce the risk of Alzheimer's. If dental professionals can use these routine tests to educate their patients and offer counseling on risk and preventative measures, more ways may be found to reduce the human suffering caused by AD. Even simply good oral health can have a positive effect on brain health [22]. This perspective focuses on the work that dental professionals regularly administer in the areas of cardiovascular health, smoking, sleep, inflammatory pathogens, and diabetes that can also serve in the prevention of Alzheimer's, with a special note on the benefit of Medicare spending in the dental field and Alzheimer's prevention.

## Health Issues Affecting Alzheimer's Disease

### Cardiovascular Health

Many factors related to cardiovascular health increase the risks of Alzheimer's [23, 24]. Midlife obesity, hypertension, and high cholesterol are all associated with an increased risk of dementia [25–27]. A study that followed Americans from 1987 to 2017 revealed that individuals with hypertension in midlife had a significant risk factor for cognitive decline [28]. In a 2014 study, Dr. Gottesman et al. found hypertension was associated with 39% greater odds of dementia [29]. Another study of over 500 people born the same week in 1946 measured blood pressure, cognitive assessments, and PET imaging at ages 36, 43, 53, 60–64, and 69 years. Results showed that elevated blood pressure in middle age resulted in drastic increases in dementia later in life, smaller brain volume, and increased blood vessel damage [30].

### Discussion

Taking blood pressure is considered standard of care in dentistry. On every patient at every visit blood pressure is taken and recorded, which enables clinicians to see subtle increases that may indicate systemic issues. Dental providers see patients as often as four times more per year than their general practitioners. Panoramic radiographs are taken during regular assessments by most dental practices at initial visits, and every 3–5 years following. The radiographs allow practitioners to see cysts, tumors, wisdom teeth, the temporomandibular joint, and bone abnormalities, including bone loss from periodontal disease. The film can also detect any calcifications in the soft tissue area. Atherosclerotic lesions of the carotid artery occur at the bifurcation of the common carotid. When these lesions are calcified, they may present on panoramic radiographs as nodular calcifications or vertical radiopacities [31]. Often, these issues remain undetected, as they rarely produce symptoms. Ultimately, the interruption of blood flow to the brain causes a loss of neurological function and may lead to an ischemic or hemorrhagic stroke. Stroke is a strong, independent, and potentially modifiable risk factor for all-cause dementia [32]. People who have had a stroke have nearly double the risk of developing Alzheimer's disease [33]. Clinicians should be careful to evaluate panoramic radiographs in patients who have a history of cardiovascular disease, and other risk factors such as smoking, hypercholesteremia, being overweight or having diabetes. Calcifications are also associated with oral infections and increased mortality and clinicians should carefully assess panoramic radiographs to intercept calcified atherosclerotic lesions of the carotid artery [34]. By remaining vigilant while reviewing radiographs, dental professionals can help identify potential risk factors that may be early indicators of Alzheimer's.

### Smoking

Smoking is linked to vascular problems, which is a contributor to Alzheimer's. This may be by way of strokes or smaller brain bleeds, but toxins in cigarette smoke increase oxidative stress and inflammation, which have been linked to Alzheimer's. A 2017 study found that a smoking habit increases the chance of dementia later in life [29]. Studies have found that smokers have reduced brain size, specifically in the cortex, which is responsible for many mental tasks including visual processing and complex abstract thinking [35]. Smoking is a leading cause of preventable death [36], so many smokers may actually die before they reach the age when dementia develops. Therefore, it is difficult for data to be conclusive as to how severely smoking is related to AD, but we know there is a strong correlation [37]. Fortunately, by quitting smoking, damage can at least be partially reversed [36].

### Discussion

Smoking cessation programs within the dental practice are already being done, lending itself to individualized support. Dental practitioners have the availability to follow the 5 A's smoking cessation plan (ask, advise, assess, assist and arrange) developed by the US Public Health Service to enhance motivation for smokers to change their behavior [38]. Whether clinicians refer services, write a prescription, or perhaps even assess current smoking status by measuring exhaled carbon monoxide levels, they have the potential to change the lives of smoking patients. Through support, care, and education dental professionals can help to illustrate to patients the dangers of smoking concerning risk of AD.

### Sleep

Sleep is critical to overall wellness [39, 40]. It helps to refresh the brain, clean out waste products [41], and replenishes nutrients. Impaired sleep effects the glymphatic system, the system that clears the brains waste products, and new research suggest that there may be a causal relationship between inadequate sleep and dementias [42]. Sleep cleans the hippocampus which facilitates consolidation of memories, which is a crucial step in being able to take in and process new information [43]. Getting proper amounts of sleep can help with most systemic ailments, from lowering blood pressure, reducing depression, obesity, regulating blood sugar, and strengthening the immune system. Proper sleep has been shown to increase amyloid clearance from the brain, and new research shows that people getting proper sleep make less amyloid [44–46]. Other studies have found that a lack of deep sleep is associated with higher tau levels, which forms toxic tangles inside brain cells and may be linked to Alzheimer's [47].

Sleep apnea, a condition that happens when the airway becomes blocked or collapses during the night, has been associated with numerous contributory factors for AD [48, 49]. According to the American Sleep Apnea Association, about 22 million Americans have sleep apnea [50]. A 2013 study found that older adults who experience fragmented sleep, such as that caused by sleep apnea, are more prone to develop Alzheimer's disease [13].

### Discussion

Dentistry has become a force to help patients with sleep apnea Dental professional's contribution to patient care in this area should include assisting in diagnosing and to referring to other healthcare specialists, and to determining when dental therapies are of value, e.g., and even treating mild to moderate apnea with mandibular advancement devices [51, 52]. The collaboration between dentists and sleep physicians is growing stronger by the day. Being able to execute simple questionnaires like the STOP Bang and the Epworth Sleepiness Scale can help identify potential risk factors. Additionally, indicators of sleep disordered breathing, such as a scalloped tongue, large tonsils, a red beaten uvula, or grinding patterns [53], can be noted with a simple cursory look during an oral cancer exam. Two of the quickest and most simple assessments are the Malampatti score and tonsil grading that are incredible evaluators and teaching tools for the patient. Dental professionals are educators and pivotal in the discussion of risks, referrals, and treatment options of disordered sleep, which may help to lower patient's risk of developing Alzheimer's disease [54, 55].

### Inflammatory Pathogens

Compelling active research regarding microbes, the immune system and the oral cavity and their interaction with inflammation keeps periodontal disease at the forefront of systemic discussions [56]. Many studies have shown that older people with periodontal disease may have an increased risk of Alzheimer's [57–59]. Inflammation causes neural damage from the cascade of events of the central nervous system starting with pro-inflammatory cytokines such as C-reactive proteins, tumor necrosis factor-a, interleukin-1 and interleukin-6 [60]. Periodontal pathogens such as Porphyromonas gingivalis, Treponema denticola, Prevotella intermedia, and even HSV1 (cold sores) may all be implicated in Alzheimer's [61–64].

Dr. Judith Miklossy has been studying spirochetes for 3 decades. She was one of the first to demonstrate spirochetes in brain matter. *In vivo* and *in vitro* spirochetes were able to reproduce the clinical, pathological and biological hallmarks of AD. These results fulfilled Hill's criteria and confirmed a causal relationship [65]. A 2021 report revealed *T. denticola* as a potential contributing factor, along with *P. gingivalis*, as risk factors for AD. The research group found that *T. denticola* had the ability to enter the brain and increased the expression of amyloid-β (the hallmark in AD), although the mechanism is unclear [66].

It has been reported that amyloid forms almost instantly around viruses and bacteria, and that infections, including mild ones that produce only minimal symptoms, fire up the immune system in the brain and leave a debris trail that is the hallmark for Alzheimer's [67, 68]. When a virus or bacterium sneaks past the blood-brain barrier, which becomes leaky with age, the brain's defense system is triggered. To combat the intruder, the brain makes amyloid to act as a sticky web to trap the intruder. The beta-amyloid is an antimicrobial peptide (basically, a protein) that the immune system creates to physically trap germs, so what is left is a webby plaque seen in the brains of those with Alzheimer's [69]. Some Alzheimer's researchers are convinced that microbes are causative in Alzheimer's [70, 71]. *P. gingivalis* is a high risk, red complex periodontal pathogen [72]. There is increasing evidence implicating the polymicrobial infection with *P. gingivalis* as playing a part in disease pathogenesis, not only from the low-grade inflammation but the actual translocation into the brain vasculature [64]. A link between Alzheimer's and *P. gingivalis* in the brain is consistent with this emerging model for microbes in the disease's etiology [58, 73–75].

### Discussion

There is mounting evidence of periodontal disease impacting altering, systemic health negatively. There are independent associations between periodontal disease and a host of systemic diseases including but not limited to: diabetes, cardiovascular diseases, certain cancers, and cognitive diseases [76, 77]. The activities in health and disease within the gut, the brain and the oral cavity seem to have overlapping consequences. For example, individuals with chronic local inflammation due to periodontal disease have corresponding changes within the gut, to include a link to inflammatory bowel disease [78]. A growing body of clinical and experimental evidence suggests that gut microbiota may contribute to and influence brain disorders [79].

Treating patient's periodontal disease and preventing future events has long been a goal of dental professionals. The strong evidence that reducing oral inflammation, created by infections of the periodontia, contributes to a reduction in systemic inflammation, reinforces the concept that dental providers have a responsibility to help in treating a patient's total health, which in turn may reduce the possibility of many systemic diseases including Alzheimer's. We are the only licensed professionals with this capability, so it is essential to educate ourselves and our patients about Alzheimer's related risks and their periodontal health. Whether they are a causal agent, a contributory agent, or an exacerbator matters not, dentistry helps communities lower their risk. Our tools, from chairside pathogen testing to the use of ozone and lasers, not to be out done by education skills and behavior change strategies, can be put to use in ways to mitigate yet another disease.

### Diabetes

Nearly 36% of all U.S. adults and 50% of those 60 years or older are estimated to have metabolic syndrome, a combination of health conditions such as obesity, high blood pressure, insulin resistance, type 2 diabetes, or a poor lipid profile [80]. The correlation between diabetes and risk for Alzheimer's has been the focus of numerous studies [81, 82]. Additionally, there is a new type of diabetes being studied, type 3, which is also strongly correlated with Alzheimer's [83]. People with type 2 diabetes may be twice as likely to develop Alzheimer's, and those with prediabetes or metabolic syndrome may have an increased risk for having predementia or mild cognitive impairment [84]. Those with high blood sugar, whether technically diabetic or not, have a faster cognitive decline rate than those with normal blood sugar [85].

### Discussion

Oral health professionals have long been aware of the complications of hypoglycemia in patients with diabetes. Many oral health professionals scrutinize health histories, question patients, and check their blood glucose levels, yet there is a need to increase the monitoring of diabetes during dental visits. In 2018, the American Dental Association added CDT code D0411 for chairside HbA1c, and in 2019 code D0412 to check blood glucose levels. This was not put in place for the expectation to diagnose diabetes, but to help monitor patients during dental procedures. Chairside screening and referral may improve prediabetes and diabetes diagnosis. Point of care testing, specifically in dental practices, can play a vital role in the early detection of type 2 diabetes [86]. Many individuals may not seek diabetic testing due to fear. Dental practitioners often have strong, trusting relationships with patients that may help them feel more comfortable discussing health concerns, like diabetes and Alzheimer's. These are incredible opportunities for dental professionals to help in early detection.

### Special Note on Medicare

Having access to dental care is difficult for many US populations especially the elderly and not having access is associated with cognitive decline [87]. It would be kinder and fiscally more responsible to extend Medicare benefits to dentistry if it lowers the cases of a disease projected to affect so many people in such a near future. In 2020, Alzheimer's and other dementias cost the nation ~$305 and $206 billion in Medicare and Medicaid payments [2]. If dental care were added to Medicare, it may help to reduce the flood of cases. Unless a treatment to slow, stop or prevent this disease is developed, in 2050, Alzheimer's cost is projected to exceed $1.1 trillion. This dramatic rise includes more than 4-fold increases in government spending under Medicare and Medicaid, and in out-of-pocket spending [2].

Medicare beneficiaries with Alzheimer's regularly have other chronic conditions, such as heart disease, diabetes, and kidney disease, and require more skilled nursing, home health, and hospital stays per year than other older people [2]. Not only are there regulations as to whether dental professionals can even do services in a care facility depending on state governing bodies, but there are minimal state and federal guidelines regarding dental care. The inclusion of dental services in Medicare may help to combat the ravages of Alzheimer's disease and curtail the rapid rise in diagnosis rates.

## Conclusion

Successful healthcare is not just the proper management of disease, but it includes strong prevention strategies. Something must be done to spread knowledge on preventive measures to help slow down the rate of Alzheimer's disease. The goal of this perspective piece is to provide examples for the need to bring dentistry into the fold of interdisciplinary approaches in healthcare. The utilization of *all* healthcare providers is essential along with universal education, partnerships, and healthcare coverage. Many of our communities are left behind due to access and cost and many professionals are not willing to serve those regions. Dental professionals focus on disease prevention, health promotion and often form long-term relationships with their patients. The nature of these relationships can help dental professionals notice subtle changes that may be indicators of a future Alzheimer's diagnosis, and preventative care and education can then be advised. New roles are emerging to help address specific treatment needs and access to dental care, but much more must be done. Many dental hygienists are open to not only expanding services into the underserved communities but working within other healthcare areas such as hospitals and physicians' offices. Including dental care coverage to Medicare would be advantageous but making needed changes will not be easy. Innovative collaborative efforts must be made, and dental professionals are uniquely situated to help roll back the tide of this horrible disease.

For further information you may be interested in attend consensus conferences addressing oral systemic health, collaboration and integration principles. Some examples include, but not limited to, The Santa Fe Group *Oral Health into Overall Health* https://santafegroup.org/events/ The American Academy of Oral Systemic Health *Collaboration Cures* https://www.aaosh.org/2021-scientific-session The Dental Integration Conference *A Time That's Come* https://www.dentalintegrationconference.com

## Data Availability Statement

The original contributions presented in the study are included in the article/supplementary material, further inquiries can be directed to the corresponding author/s.

## Author Contributions

AOR conceived and presented the idea and wrote the perspective article based upon their opinion and data supporting that opinion.

## Conflict of Interest

AOR is the owner of company Oral Systemic Seminars.
